# The relations among depressive symptoms, self-esteem, and optimism during adolescence: Longitudinal evidence from nine countries

**DOI:** 10.1017/S0954579425100497

**Published:** 2025-09-15

**Authors:** Chelsea Cortright, Danming An, Jennifer E. Lansford, Marc H. Bornstein, Lei Chang, Kirby Deater-Deckard, Laura Di Giunta, Kenneth A. Dodge, Sevtap Gurdal, Daranee Junla, Qin Liu, Qian Long, Paul Oburu, Concetta Pastorelli, Ann T. Skinner, Emma Sorbring, Laurence Steinberg, Liliana Maria Uribe Tirado, Saengduean Yotanyamaneewong, Liane P. Alampay, Suha M. Al-Hassan, Dario Bacchini

**Affiliations:** 1 Department of Psychology, Lehigh University, Bethlehem, PA, USA; 2 Center for Child & Family Policy, Duke University, Durham, NC, USA; 3 Sanford School of Public Policy, Duke University, Durham, NC, USA; 4 Eunice Kennedy Shriver National Institute of Child Health and Human Development, Bethesda, MD, USA; 5 Institute for Fiscal Studies, London, UK; 6 UNICEF, New York City, NY, USA; 7 Department of Psychology, University of Macau, Macau, China; 8 Department of Psychological and Brain Sciences, University of Massachusetts Amherst, Amherst, MA, USA; 9 Department of Psychology, Università di Roma “La Sapienza,”, Rome, Italy; 10 Division of Educational Science and Languages, University West, Trollhättan, Sweden; 11 Division of Social Work and Social Pedagogy, University West, Trollhättan, Sweden; 12 Department of Psychology, Chiang Mai University, Chiang Mai, Thailand; 13 Department of Maternal and Child Health & Adolescent Health, Chongqing Medical University, Chongqing, China; 14 Department of Global Health, Duke Kunshan University, Kunshan, China; 15 School of Education, Maseno University, Maseno, Kenya; 16 Department of Psychology and Neuroscience, Temple University, Philadelphia, PA, USA; 17 Center for Social and Humanities Research, King Abdulaziz University, Jeddah, Saudi Arabia; 18 Universidad de San Buenaventura, Medellín, Colombia; 19 Department of Psychology, Ateneo de Manila University, Quezon City, Philippines; 20 Abu Dhabi Early Childhood Authority, Abu Dhabi, United Arab Emirates; 21 Department of Humanistic Studies, University of Naples “Federico II,”, Naples, Italy

**Keywords:** Adolescence, cross-cultural, depressive symptoms, optimism, self-esteem

## Abstract

Previous research has suggested bidirectional relations between depressive symptoms and both internal and external core beliefs (self-esteem and optimism, respectively) in adolescence. However, little work has examined the cultural commonality versus specificity of these developmental pathways in adolescence across diverse contexts. To address this gap, the current study traced bidirectional associations among depressive symptoms and two forms of core beliefs (self-esteem and optimism) in adolescents from 12 cultural groups in nine countries. Longitudinal data were collected from 1,090 adolescents at ages 15 and 17. Significant associations emerged between age 15 depressive symptoms and both age 17 core beliefs across all cultural groups except Sweden. No significant associations between age 15 core beliefs and age 17 depressive symptoms were found in the multigroup model. However, the pathways from core beliefs to depressive symptoms and from depressive symptoms to core beliefs did not significantly differ in strength. These findings provide cross-cultural evidence for the scar theory (depressive symptoms → core beliefs), but no clear support for the vulnerability theory (core beliefs → depressive symptoms), perhaps due to the measurement and stability of depression. These findings have implications for understanding the adolescent development of psychopathology and cognitions, such as core beliefs, across diverse cultures.

## Introduction

Depression is one of the leading causes of illness and disability in adolescence and is experienced by an estimated 3.9% of the global adolescent population (World Health Organization, 2021). The significant prevalence of depression during adolescence is attributed to several factors, such as the biological and social changes which adolescents experience, as well as their cognitive maturation and identity development (Thapar et al., 2012). Compared with children, adolescents are more self-aware and increasingly capable of complex and abstract thinking about themselves and the social world, embarking on a journey of self-discovery where they explore and formulate their personal identity (Erikson, 1968). This pronounced awareness and evaluation of the self and the social world can be associated with negative beliefs and life distress, which are notably involved in the development of depression (Garber et al., 1993; Hovenkamp-Hermelink et al., 2019; Ohannessian et al., 2018; Tak et al., 2017).

Theories about relations between core beliefs and depression have asserted reciprocal links between the two constructs. *Vulnerability theory* (Beck, 1967; Ingram et al., 1998) suggests that negative core beliefs about oneself and the world give rise to the onset and maintenance of depression. Complementarily, *scar theory* emphasizes that experiencing depression may cause an individual to perceive things more negatively, and in turn, adversely affects their core beliefs (Lewinsohn et al., 1981). Both the vulnerability and scar theory have received some support from empirical studies in the adolescent population (Gittins & Hunt, 2020; Orth et al., 2012; Peñate et al., 2020; Saint-Georges & Vaillancourt, 2020; Trzesniewski et al., 2006), with some research suggesting that the pathways may co-occur (Johnson et al., 2016). However, the evidence for co-occurrence is not fully consistent, and few studies of adolescents have examined these bidirectional pathways simultaneously.

Additionally, research on depression and core beliefs during adolescence has primarily focused on *internally focused core beliefs* such as self-esteem, which can be defined as “the self-evaluation and descriptive conceptualization that individuals make and maintain with regard to themselves” (Abdel-Khalek, 2016, p. 3, Gruenenfelder-Steiger et al., 2015; Masselink, 2018; Rosenberg et al., 1989). Less work has been conducted on the relation between depression and *externally focused core beliefs*, such as optimism. Optimism, which can be defined as “hopefulness and confidence about the future, a tendency to take a favorable view of things, and an explanatory style marked by evaluating negative events as temporary, external and specific to situations” (Kern et al., 2016, p. 587), is conceptually associated with depression yet is less well understood in terms of its co-evolvement with depression during adolescence (Cheavens, 2000; Tao, 2006). Such internally and externally focused core beliefs are conceptually and empirically related constructs, which are considered facets under the umbrella construct of positive orientation (Caprara et al., 2010). However, those two facets have often shown different associations with mental health and psychological well-being, which warrants further exploration of their individual unique relations to psychopathology (Caprara et al., 2010; Nawa & Yamagishi, 2024; Weinberg et al., 2015).

Theories on the psychopathology of depression, such as the cognitive triad model (Beck, 1967; 1976), often distinguish between internally and externally focused core beliefs (e.g., negative views about self vs. others/future) and discuss their unique roles in clinical work. For example, internally and externally focused core beliefs are driven by different cognitive schemas (Newman, 2003), and create distinct, unique risks for the onset and maintenance of depressive symptoms (Hankin et al., 2007). In addition, depression often presents with a diverse set of symptoms, encompassing cognitive, emotional, and somatic experiences. The distinct symptomatology of an individual’s experience with depression has unique relations to their subsequent core beliefs, with differing relations to beliefs of the self, of the future, and of the world (Marchetti & Possel, 2022). For these reasons, we aim to examine the relation between depressive symptoms and both internally focused (i.e., self-esteem) and externally focused (i.e., optimism) core beliefs.

### Self-esteem, optimism, and depression

Substantial research has supported negative bidirectional longitudinal associations between self-esteem and depression among adults (Sowislo & Orth, 2013). However, research examining the bidirectional longitudinal associations in adolescence has been more sparse, and the work that has been done is not conclusive. Some studies have found support for self-esteem predicting future depressive symptoms, but not vice versa (Orth et al., 2008; van Tuijl et al., 2014). By contrast, other studies have suggested that depressive symptoms predict future self-esteem but not the reverse (Ohannessian et al., 1999). Other research has even failed to find evidence for either pathway (Kakihara et al., 2010).

Another core belief that has been studied (albeit less extensively) in its longitudinal relation to depressive symptoms is the concept of optimism. Research on the relation between optimism and depressive symptoms has shown that the two constructs are concurrently related in adolescence (Kwok & Gu, 2017; Weber et al., 2010; Wong & Lim, 2009). Less longitudinal research has been conducted, however, and much of that work has focused on the effects of optimism on later depressive symptoms in adolescence (Patton et al., 2011; Smokowski et al., 2014). The literature examining the effects of depressive symptoms on later optimism has been limited to adulthood (Karhu et al., 2024; Li et al., 2018). Although this relation has been supported within adult populations, it is important to examine such relations within adolescence specifically.

Most of the aforementioned literature looking at the relation of both core beliefs (self-esteem and optimism) with depressive symptoms has been limited to minority world Western, industrialized populations, whereas adolescents from the majority world have received much less attention. However, there is both theoretical and empirical support for potential differences in these relations across cultures.

### Cross-cultural similarities and differences in the relations between core beliefs and depressive symptoms

Although prevalence rates vary, depression is an affliction experienced globally (Moreno-Agostino et al., 2021; Shorey et al., 2022). Research supports some similarities in depression across diverse contexts. For example, some factors, such as negative life events and lack of adequate coping strategies, are a risk for elevated depressive symptoms in adolescence across cultures (Auerbach et al., 2010). Further, cross-cultural comparison studies as well as meta-analytic work has supported that cognitive schema, including core beliefs, are associated with depression across cultures (Bartucz et al., 2022; Mahali et al., 2020; Sowislo & Orth, 2013).

However, there are also cross-cultural differences in the manifestation of depression. One cultural difference often emphasized is the distinction between individualism and collectivism. Individualistic cultures (typically Western countries) tend to emphasize an independent self-construal, which focuses on being distinct, unique, and separate from others. By contrast, collectivistic cultures (typically Eastern countries) often foster an interdependent self-construal, which emphasizes connectedness and interrelations with others (Markus & Kitayama, 1991). Some studies suggest that collectivistic cultural values may serve as a general protective factor that mitigates the association between negative cognitive beliefs and depression due to the emphasis on interpersonal relationships and supportive networks (Bartucz et al., 2022). Further, due to varying self-construals across individualistic and collectivistic cultures, core beliefs that focus on the internal self, versus the external world, may also vary in their salience across cultural contexts (Chiles et al., 1989; Chung & Mallery, 1999), especially in terms of their associations with psychological symptoms and well-being (Assari & Lankarani, 2016; Chang et al., 2003; Chang, 1996; Eshun, 1999; Titova et al., 2017). Most studies supporting this notion, though, have focused on the concurrent relations between core beliefs and well-being within adult populations. Only one study to our knowledge has inspected the longitudinal relations between both internally and externally focused core beliefs and depressive symptoms across cultures in an adolescent sample. Stewart and colleagues (2004) examined the bidirectional relations among hopelessness, self-efficacy, and depression in adolescents within China and the United States. They found support for bidirectional pathways in both countries, but with some notable differences. Specifically, the core beliefs (particularly self-efficacy) showed weaker concurrent and longitudinal effects on depression in the Chinese sample compared to the U.S. sample, potentially because the independent self is emphasized less in China. In addition, two large multi-national studies that examined the concurrent associations between internally focused core beliefs and psychological well-being during adolescence also have suggested similar patterns, that the association between self-esteem/self-efficacy and psychological well-being (i.e., depression, life satisfaction) is stronger in individualistic cultures than in collectivistic cultures (Kwon & Kim, 2024; Smith et al., 2016). Thus, it appears that adolescents’ internally focused core beliefs (e.g., self-esteem) are more strongly associated with depressive symptoms in individualistic than collectivistic cultures. The associations between externally focused core beliefs (e.g., optimism) and depression among adolescents in individualistic and collectivistic cultures are less clear and warrant further exploration.

In addition to individualism and collectivism, different societies often also vary in their resources and challenges. Those resources and challenges may particularly impact how optimistic individuals in a cultural context can feel about their future. Correspondingly, cross-cultural variables, such as gross domestic product and power structure, influence average levels of optimism in each culture (Fischer & Chalmers, 2008; Joshi & Carter, 2013), which in turn, can contribute to, and be impacted by, the development of depressive symptoms. In one large cross-cultural study examining the relations between optimism and well-being across 61 countries, researchers found a positive relation between the two, but also found that this relation was much weaker in countries with more economic and social hardships (Baranski et al., 2021). It is possible, therefore, that the associations between adolescents’ optimism and depressive symptoms may vary across countries with different resources and challenges.

Further, there also appear to be cross-cultural differences in the symptomatic expression and experience of depression. For example, individuals in Western cultures may be more likely to “medicalize” their depressive symptoms, allowing them to envision their depression as an entity separate from the self (Chentsova-Dutton & Tsai, 2009). In contrast, some Eastern cultures are more likely to interpret their depressive symptoms as an interpersonal failure, rather than a medical diagnosis. Different cultures also have varying levels of stigma about mental health problems (Krendl & Pescosolido, 2020). These cultural differences on the perspectives and interpretations of symptoms may create diverse pathways in how depression relates to later core beliefs of the self and the world. In sum, although there are considerable cross-cultural similarities in the associations between core beliefs and depression, factors such as individualistic and collectivistic cultural values, societal resources and challenges, and perspectives on psychological symptoms may contribute to cross-cultural variations in the associations between core beliefs and depression among adolescents. The cross-cultural similarities and differences may also depend on the specific domain of core beliefs (e.g., self-esteem versus optimism). Very few longitudinal studies have been conducted to systematically examine the cross-cultural similarities and differences in the associations between adolescents’ core beliefs and depressive symptoms. More work is needed to explore this research question and inform interventions across the globe.

### The present study

In the current study, we aimed to comprehensively investigate the potential bidirectional relations between adolescent depressive symptoms and two core beliefs in a diverse cross-cultural sample. Our goals included 1) examining both vulnerability and scar pathways simultaneously in an adolescent sample; 2) expanding to the two distinct types of core beliefs, namely self-esteem (internally focused) and optimism (externally focused); and 3) analyzing cross-cultural commonality and uniqueness in the development of depressive symptoms and core beliefs across 12 cultural groups. We expected the findings to support both vulnerability and scar pathways (i.e., age 15 self-esteem and optimism are associated with age 17 depressive symptoms, and age 15 depressive symptoms are associated with age 17 self-esteem and optimism), but hypothesized that these associations may depend on the specific core belief variable (i.e., self-esteem vs. optimism) within specific cultural contexts. However, as the previous literature has mostly focused on self-esteem rather than optimism, and no previous work has examined the longitudinal relations between specific domains of core beliefs and depressive symptoms in adolescents across multiple cultural groups, the cross-cultural comparison part of the study is largely exploratory.

## Method

### Participants

A sample of 1,090 adolescents across nine different countries participated in our study. The data were part of a larger longitudinal study, Parenting Across Cultures. Adolescents were recruited from Medellín, Colombia (*n* = 80), Naples, Italy (*n* = 92), Rome, Italy (*n* = 104), Zarqa, Jordan (*n* = 102), Kisumu, Kenya (*n* = 79), Manila, Philippines (*n* = 92), Trollhättan/Vänersborg, Sweden (*n* = 85), Chiang Mai, Thailand (*n* = 90), Chongqing, China (*n* = 114), and Durham, North Carolina, USA (*n* = 252). These sample sizes reflect the overall sample at each site at Time 1 of the present study. However, due to missing data on some variables as well as attrition across Time 1 and Time 2, these sample sizes vary by time and variable. Please refer to Supplementary Tables 1-12 for the sample size for all variables at all sites across both time points.

Most of the included sites were ethnically homogeneous. However, two of our sites (USA and Italy) had some differences within the sample. Specifically, the USA sample included three diverse ethnic groups (i.e., Latino American, *n* = 70; African American, *n* = 89; and European American, *n* = 93). Italy included two diverse regions (Naples, *n* = 89 and Rome, *n* = 102). Research supports distinct cultural differences across these groups (Martinez-Fuentes et al., 2020; Rothenberg et al., 2020) and thus, we kept the two Italian groups, as well as the ethnic groups in the USA sample, separate in our analysis. This resulted in 12 separate groups in our analysis. For further descriptive statistics on the samples, refer to Table 1.

**Table 1.** Descriptive statistics and zero-order correlations among study variables across all 12 groups in nine countries

	1	2	3	4	5	6	7	8
1. Age 15 Depressive Symptoms	–	−.47***	−.29***	.54***	−.30***	−.22***	.24***	.05
2. Age 15 Self-Esteem		–	.57***	−.35***	.51***	.39***	−.05	−.01
3. Age 15 Optimism			–	−.23***	.40***	.51***	−.02	−.04
4. Age 17 Depressive Symptoms				–	−.51***	−.35***	.24***	.07*
5. Age 17 Self-Esteem					–	.60***	.02	−.02
6. Age 17 Optimism						–	.01	−.05
7. Gender							–	.01
8. Highest Parental Education								–
*N*	1079	1039	1041	957	964	961	1088	897
Mean	0.46	3.91	3.40	0.56	3.89	3.49	1.51	13.96
SD	0.42	0.88	0.99	0.46	0.90	1.03	0.50	4.51
Minimum	0.00	1.00	1.00	0.00	1.00	1.00	1.00	0.00
Maximum	2.00	5.00	5.00	2.00	5.00	5.00	2.00	30.00
Skewness	0.98	−0.62	−0.12	0.63	−0.69	−0.32	−0.06	0.13
Kurtosis	0.60	−0.15	−0.91	−0.42	0.13	−0.78	−2.00	0.40

Notes.*p ≤ .05. **p ≤ .01. ***p ≤ .001. Gender was coded with a 1 indicating boy, and 2 indicating girl.

### Procedures

Adolescents and their families were recruited from schools selected to represent the socioeconomic diversity within each country. Letters were sent home from schools (both public and private, serving high-, middle-, and low-income families) with the children when they were 8 years old. Across most sites, the participants recruited were representative of the majority ethnic and racial group for that site. The exceptions to this are in Kenya, in which adolescents recruited were all members of the Luo ethnic group, and in the United States (as described above). Refer to Supplemental Materials Tables 1 through 12 for demographic characteristics of the sample by country. At age 15 and age 17, adolescents completed questionnaires regarding their depressive symptoms, self-esteem, and optimism.

The multinational research team evaluated the questionnaire items for their cultural appropriateness and developed translations for each country using forward and backward translation and cultural adaptation procedures. Adolescents were given the option of completing questionnaires via an oral interview, on paper, or online. Questionnaires were completed at the participant’s school, home, or other location selected by the participants and their families. Participants were given small incentives for their participation, including small monetary incentives, entries into prize drawings, or school donations, as deemed appropriate by local IRBs.

The study was approved by the Duke University IRB as well as IRBs in all the participating countries. All parents of the adolescents completed signed informed consent statements. For more information about participants and sampling procedures at each site, visit the study website at: https://parentingacrosscultures.org/.

### Measures

#### Depressive symptoms

Adolescents completed the Youth Self Report of the Child Behavior Checklist (CBCL; Achenbach, 1999). The Checklist captures various internalizing and externalizing behavioral symptoms. The composite score of depressive symptoms was formed using the mean of six items from the internalizing subscale. These six items encompassed physical (e.g., “I don’t have much energy”), cognitive (e.g., “I feel that no one loves me”), and emotional depressive symptoms (e.g., “I am unhappy, sad or depressed”). To provide an accurate examination of the bidirectional associations between depressive symptoms and self-esteem/optimism, for the cognitive symptoms, we intentionally excluded items that conceptually overlap with self-esteem or optimism (e.g., “feeling worthless”). Items were rated as 0 = *not true*, 1 = *somewhat or sometimes true*, and 2 = *very true or often true*. Internal consistency reliabilities across the countries were .79 at age 15 and .80 at age 17. Refer to Supplementary Material Table 13 for reliabilities within countries.

#### Self-esteem

Adolescents completed the 8-item Views About Life measure (Caprara et al., 2012) which assessed their evaluations of their current period of life. The self-esteem composite was computed using three items from this measure: “On the whole, I am satisfied with myself,” “I feel I have many things to be proud of,” and “I generally feel confident in myself.” The items were rated on a 5-point scale ranging from 1 = *strongly disagree* to 5 = *strongly agree*. Internal consistency reliabilities across the countries were .83 at age 15 and .83 at age 17. Refer to Supplementary Table 13 for reliabilities within countries.

#### Optimism

Adolescents completed the EPOCH measure of adolescent well-being (Kern et al., 2016) which assessed their positive adjustment and well-being. Optimism was measured using the Optimism subscale, which consisted of 4 items (e.g., “In uncertain times, I expect the best,” “I think good things are going to happen to me”). The items were rated on a 5-point scale ranging from 1 = *almost never* to 5 = *almost always*. Internal consistency reliabilities across the countries were .86 at age 15 and .87 at age 17. Refer to Supplementary Material Table 13 for reliabilities within countries.

#### Covariates: gender and parental education

Parents reported the target adolescents’ gender at the entrance of the study (age 8), and their own highest level of education (in years) when the adolescent was age 17. We included gender and parental education (as a measure of socioeconomic status) as covariates, as research often supports differences in the mean level of depressive symptoms across gender and socioeconomic status in adolescence (Devenish et al., 2017; Dyer & Wade, 2012; Lorant et al., 2003; Shorey et al., 2022).

### Analytic plan

**Figure 1.**
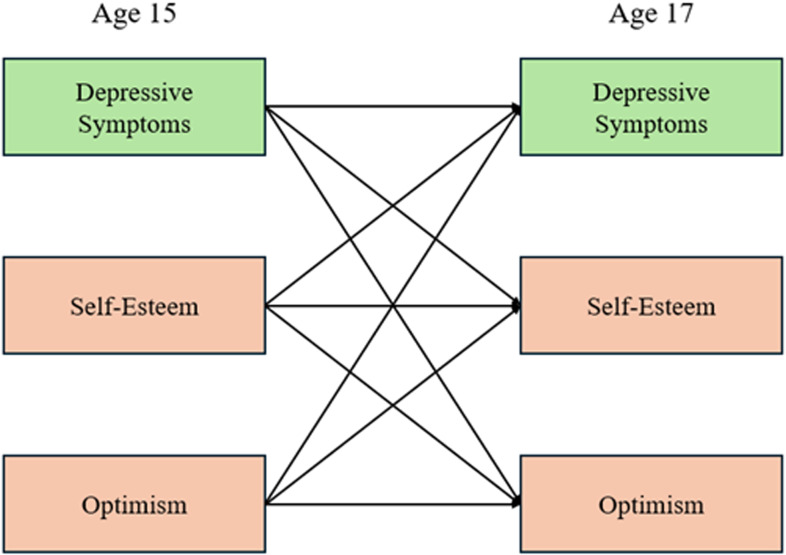
Model of bidirectional pathways between age 15 and age 17 depressive symptoms, self-esteem, and optimism. The model represents the bidirectional pathways we planned to examine in our model, with constructs of interest being entered at two time points, age 15 and age 17. The model also includes two covariates, gender, and highest parental education. Variables measured at the same time were estimated as covarying (covariances not depicted). We estimated the model in 12 cultural groups across nine countries: Medellin, Colombia (*n* = 80); Naples, Italy (*n* = 92); Rome, Italy (*n* = 104); Zarqa, Jordan (*n* = 102); Kisumu, Kenya (*n* = 79); Manila, Philippines (*n* = 92); Trollhattan/Vanersborg, Sweden (*n* = 85); Chiang Mai, Thailand (*n* = 90); Chongqing, China (*n* = 114); and North Carolina, USA (Latino American, *n* = 70; African American, *n* = 89; and European American, *n* = 93). We first ran an overall model across the 12 groups, and then conducted a multigroup analysis in which path coefficients were constrained to be the same across groups. We then freed the constraints on the path coefficients that appear different across groups based on model modification indices, until model fit became satisfactory (refer to Cheung & Rensvold, 2002 for criteria).

We conducted structural equation models using M*plus* 8.8 (Muthén & Muthén, 1998–2024). We used full information maximum likelihood to account for missing data and the robust estimator (MLR) to produce unbiased standard error estimates for non-normal data. To examine the proposed bidirectional relations, we estimated a model in which autoregressive and transactional paths were fitted between age 15 and age 17 depressive symptoms, self-esteem, and optimism. Child gender and parental education were controlled as additional exogenous variables. We also included covariances among the exogenous variables and among the age 17 endogenous variables. Refer to Figure 1 for the model configuration. We considered root mean square error of approximation (RMSEA) < .06, comparative fit index (CFI) > .95, and standardized root mean residual (SRMR) < .08 as indicators of good fit to the data (Hu & Bentler, 1999).

We first estimated this overall model across the 12 groups from the nine countries, which is a saturated model. Then, we conducted a multigroup analysis to inspect the fit of this overall model for each group by constraining the path coefficients to be equal across groups, and compared this model against a model in which all path coefficients were freely estimated. To compare the two multigroup models, following the recommendations of Cheung and Rensvold (2002), we compared their chi-squares (using Satorra-Bentler correction; Bryant & Satorra, 2012) and CFIs. A nonsignificant chi-square difference combined with a CFI difference < .01 indicated that the two models being compared did not differ in their fit to the data (Cheung & Rensvold, 2002); in other words, the constrained model fit the data well across the 12 groups in the nine countries. If these criteria were not met, it meant that the fully constrained model fit the data significantly worse than the freely estimated model; in other words, the path coefficients may differ across groups. In this case, we inspected the modification indices to find the specific path coefficient in one group, which, if allowed to be different from the other groups, would lead to the largest improvement of the chi-square. We then freed the constraint on this path, reevaluated the fit of the revised model, and iterated this process until the model fit became acceptable.

After examining bidirectional associations between depressive symptoms and self-esteem/optimism, we also compared the strengths of these pathways, as previous work has suggested that the vulnerability pathways (from core belief to depressive symptoms) may be stronger compared to the scar pathways (from depressive symptoms to later core belief; Orth & Robins, 2013). Specifically, for each country, we constrained the bidirectional paths between self-esteem and depressive symptoms to be equal; we similarly constrained the bidirectional paths between optimism and depressive symptoms. If the constraints resulted in a worse model fit, it meant that the two pathways differed in strength. Because the constructs were measured using different scales, we used the z-scores of depressive symptoms, self-esteem, and optimism for this analysis.

## Results

### Preliminary analysis

The descriptive statistics across groups can be found in Table 1. For the descriptive statistics specific to each of the 12 groups, refer to Supplemental Materials Tables 1 through 12. The mean level of depressive symptoms across groups was low for both age 15 and age 17, with means of 0.46 (SD = 0.42) and 0.56 (SD = 0.46), respectively. The mean scores of self-esteem for age 15 and age 17 remained similar across time, with means of 3.91 (SD = 0.88) and 3.89 (SD = 0.90), respectively. Similarly, optimism scores at ages 15 and 17 remained similar across time, with mean scores of 3.40 (SD = 0.99) and 3.49 (SD = 1.03), respectively. There was a wide range of parental education, ranging from 0 years (lowest level) to 30 years (highest level), with a mean of 13.96 years (SD = 4.51).

Table 1 displays the correlation matrix of all variables. The study variables showed moderate stability from age 15 to age 17, and were correlated with each other within and across the two time points. Compared with boys, girls reported significantly higher depressive symptoms at both age 15 and age 17. Correlation matrices by country are presented in the Supplementary Materials, Tables 1 through 12.

### Main analyses: bidirectional relations among depressive symptoms, self-esteem, and optimism

#### Overall model across groups and countries

Figure 1 displays our overall configural model across groups and countries. In this model, after accounting for the stability of each construct over time, several significant paths emerged. Specifically in relation to our hypotheses, a bidirectional relation between self-esteem and depressive symptoms was found. Specifically, lower self-esteem at age 15 was associated with higher depressive symptoms at age 17, and higher depressive symptoms at age 15 were associated with lower self-esteem at age 17. There were no significant relations between optimism and depressive symptoms across time points. For the covariates, girls reported higher age 17 depressive symptoms and self-esteem, but no gender difference was found for optimism. Parental education was not associated with any of the age 17 outcomes. For full results for this model, refer to Table 2.

**Table 2.** Predictions of age 17 depressive symptoms, self-esteem, and optimism from the same set of variables at age 15 and covariates

		1. Overall Model across Groups and Countries					2. Final Multigroup Model by Groups and Countries^a^		
DV	Predictor	*B*	SE	*p*	*R* ^*2*^		*B*	SE	*p*
Age 17 Depressive Symptoms	Age 15 Depressive Symptoms	**0.478**	**0.037**	**< .001**			**0.476**	**0.035**	**< .001**
	Age 15 Self-Esteem	**−0.064**	**0.020**	**.001**			−0.029	0.021	.162
	Age 15 Optimism	−0.014	0.016	.369			−0.027	0.016	.099
	Gender (1=Boy, 2=Girl)	**0.116**	**0.026**	**< .001**			**0.106**	**0.025**	**< .001**
							**(0.248**	**0.076**	**.001)** ^**a**^
	Parental Education	0.004	0.003	.147			0.003	0.003	.265
					**.324**				
Age 17 Self-Esteem	Age 15 Depressive Symptoms	**−0.185**	**0.076**	**.015**			**−0.190**	**0.073**	**.009**
	Age 15 Self-Esteem	**0.404**	**0.046**	**< .001**			**0.333**	**0.047**	**< .001**
	Age 15 Optimism	**0.134**	**0.033**	**< .001**			**0.121**	**0.033**	**< .001**
	Gender (1=Boy, 2=Girl)	**0.119**	**0.051**	**.020**			0.056	0.051	.279
							**(0.697**	**0.154**	**< .001)** ^**a**^
	Parental Education	−0.001	0.006	.866			0.003	0.006	.641
					**.294**				
Age 17 Optimism	Age 15 Depressive Symptoms	−0.125	0.087	.149			**−0.202**	**0.081**	**.013**
							(0.254	0.161	.115)^b^
	Age 15 Self-Esteem	**0.150**	**0.048**	**.002**			**0.122**	**0.047**	**.009**
	Age 15 Optimism	**0.450**	**0.037**	**< .001**			**0.333**	**0.040**	**< .001**
							**(0.553**	**0.084**	**< .001)** ^**c**^
	Gender (1=Boy, 2=Girl)	0.086	0.059	.144			0.033	0.056	.552
							**(0.578**	**0.190**	**.002)** ^**a**^
	Parental Education	−0.006	0.007	.354			0.005	0.007	.507
					**.290**		**(−0.045**	**0.014**	**.001)** ^d^

*Notes. B* = Unstandardized path coefficient. SE = Standard error of the unstandardized coefficient. We present the final multigroup model in the table, which fit the data well: χ^2^(159) = 177.85, *p* = .15; CFI = .992; RMSEA = .036, SRMR = .10. Refer to Figure 1 for the model setup and the methodology for estimating the multigroup model. We report the country/group-invariant paths and put country/group-specific paths in the parentheses with the country/group noted. Significant findings are bolded. ^a^Jordan. ^b^Sweden. ^c^Rome, Italy. ^d^Philippines. Refer to Supplementary Materials Table 14 for R^2^s by country/group.

#### Multigroup model across groups and countries

Next, we examined whether the aforementioned overall model fit across the 12 groups in nine countries. We did so by comparing a freely estimated multigroup model with a model in which the path coefficients were constrained as equal across the 12 groups. The fully constrained model fit the data significantly worse than the freely estimated model, ∆S-B χ^2^(165) = 225.921, *p* = .001, ∆CFI = .025, indicating that path coefficients were not invariant across the 12 groups. Following the procedures in the analytic plan, we released six path coefficients until an acceptable model fit was reached, ∆S-B χ^2^(159) = 177.853, *p* = .146, ∆CFI = .008 as compared with the freely estimated multigroup model.

Refer to Table 2 for a complete list of path coefficient estimates in the partially constrained multigroup model. The partially constrained multigroup model revealed similar findings as the overall model, with a few exceptions. In general, the multigroup model seemed to support the scar, but not the vulnerability, pathways. Specifically, for self-esteem, higher depressive symptoms at age 15 were still associated with lower self-esteem at age 17, but there was no significant relation between age 15 self-esteem and age 17 depressive symptoms. For optimism, we also observed a similar pattern, that age 15 depressive symptoms were associated with lower optimism at age 17 in most countries, but not the other way around.

In our final multigroup model, we also identified six paths that differed across groups. Specifically, one transactional path differed across groups: For most groups, higher depressive symptoms at age 15 were associated with lower optimism at age 17. However, this relation was nonsignificant for the sample in Sweden.

In addition, we found that four paths from the covariates to the age 17 outcomes differed across groups. Across most groups, gender was not significantly associated with age 17 self-esteem or optimism. However, in the sample from Jordan, gender was significantly associated with these outcomes, with girls reporting significantly higher levels of both. Also, across all groups gender was associated with age 17 depressive symptoms, with girls reporting higher symptoms. However, the effect size for this pathway was significantly larger in Jordan. In addition, parental education was not associated with any age 17 outcomes in most groups. However, surprisingly, higher parental education was associated with *lower* optimism in the sample from the Philippines. The findings about gender and parental education were consistent with the country-specific zero-order correlations.

**Figure 2.**
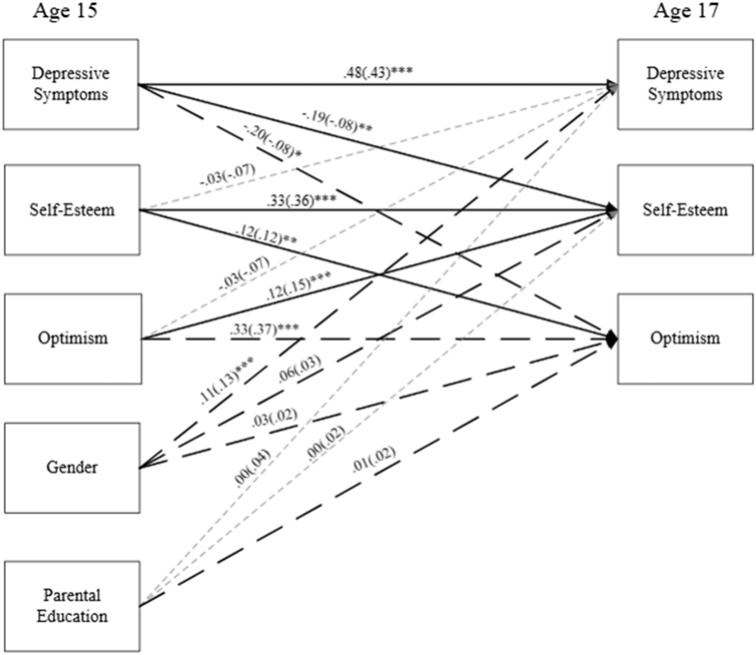
Bidirectional pathways among age 15 and age 17 depressive symptoms, self-esteem, and optimism across the 12 groups in nine countries. The model depicts the common pattern of path coefficients across the 12 groups in the final multigroup model. Group-specific pathways that deviate from this common pattern are reported in Table 2. We report both unstandardized and standardized (in parentheses) coefficients. Bold, solid lines indicate group-invariant significant pathways. Un-bolded, dashed lines indicate group-invariant nonsignificant pathways. Bold, dashed lines indicate pathways that differ across groups. *p < .05. ** *p* < .01. ***p < .001.

Finally, the last pathway that differed across groups concerned the stability of optimism from age 15 to age 17. Specifically, the stability in optimism from age 15 to age 17 was significantly higher in Rome than in the other samples. See Figure 2 for a summary of all significant and nonsignificant pathways in the final multigroup model.

#### Comparing the vulnerability and scar pathways

To examine whether the vulnerability vs. scar pathways have equal strengths, we constrained the standardized coefficients of each pair of such pathways to be equal (i.e., self-esteem → depressive symptoms = depressive symptoms → self-esteem, and optimism → depressive symptoms = depressive symptoms → optimism). We applied the same constraints to all 11 groups (except for Sweden, due to their country-specific pattern of the nonsignificant pathway from depressive symptoms to optimism). Constraining the vulnerability and scar pathways to be the same did not change the fit of the data, ∆S-B χ^2^(2) = 4.059, *p* = .13, ∆CFI = -.003. Therefore, we found no support for differences in strength across the scar and vulnerability pathways.

## Discussion

Depressive symptoms and negative, maladaptive core beliefs of the self and the world are often intertwined with each other. The links between depression and core beliefs can be bidirectional – pessimistic perceptions of the self and the world may increase the risk of developing depressive symptoms (i.e., the vulnerability theory), and depressive symptoms, in turn, may change one’s core beliefs leading to more negative thoughts and perceptions of the self and the world (i.e., the scar theory). Most existing studies examining these pathways in adolescence have been conducted in Western, industrialized countries. In addition, most adolescent studies have focused on the relations between depressive symptoms and internally focused core beliefs, such as self-esteem. Limited work has examined the longitudinal relation between depressive symptoms and other externally focused core beliefs, such as optimism.

In the present study, we examined both the vulnerability and the scar hypotheses among multiple cultural groups of adolescents using longitudinal data. Furthermore, we extended the hypotheses to examine associations between depressive symptoms and core beliefs regarding the self (internal; i.e., self-esteem) as well as the surrounding context (external; i.e., optimism). The findings supported mostly similarities across cultures but also revealed some cross-cultural differences. Below, we discuss the culturally common findings first, and then the culturally specific findings.

### Cross-cultural similarities in the associations between core beliefs and depressive symptoms

Our findings provide strong support for the scar theory (depressive symptoms → core beliefs) for both self-esteem and optimism. Across the various cultural groups, higher depressive symptoms at age 15 were associated with lower self-esteem at age 17. We observed the same association between depressive symptoms and later optimism in most cultural groups, except for Sweden. Therefore, in most countries, it appears that experiencing elevated depressive symptoms may leave a “scar” on the adolescents’ internal and external core beliefs, impacting perceptions of both the self and the world. Our findings among adolescents regarding the scar pathways are largely consistent with the adult literature (Karhu et al., 2024; Li et al., 2018; Ohannessian, 1999; Orth et al., 2008).

The scar pathways have important implications for interventions. Because depressive symptoms are associated with later negative internal and external core beliefs, specific attention should be paid to using interventions that bolster *both* types of core beliefs in adolescents who are demonstrating elevated depressive symptomatology. Although self-esteem and optimism are both enhanced via therapeutic interventions, each core belief may respond differently to various therapeutic approaches (Moloud et al., 2022). Ensuring that adolescent interventions are successfully targeting both forms of core beliefs is important for maximizing treatment outcomes.

The findings from our multigroup model did not provide support for the vulnerability theory (core beliefs → depression) across the cultural groups for either self-esteem or optimism. This was somewhat surprising, given that the vulnerability theory has received ample support within the adult literature (Orth & Robins, 2013). However, the findings for the vulnerability pathway in the adolescent literature have been more mixed (Kakihara et al., 2010; Ohannessian, 1999; Orth et al., 2008). Considering that adolescence is a period marked by identity exploration and malleable core beliefs (Ditzfeld & Showers, 2013; Zou et al., 2016), it is possible that the effect of depression on the formation of core beliefs, rather than the other way around, is more salient during adolescence. While this is one possible interpretation, it is important to note that although the vulnerability pathways were nonsignificant in the multigroup model, they were not statistically different from the scar pathways in terms of the size of the standardized effects. In addition, the zero-order correlations showed that age 15 self-esteem and optimism were both significantly associated with age 17 depressive symptoms. It is possible that due to the stability of depressive symptoms during this developmental period, the autoregressive pathways have already explained much of the variance in age 17 depressive symptoms, which makes the effects of core beliefs less prominent.

It is also worth noting that our choice of items for depressive symptoms differed slightly from previous studies. Specifically, we excluded items from our depressive measure which had strong conceptual overlap with our core belief variables (e.g., feeling worthless). If we include the overlapping items back in the analysis, the findings support a vulnerability pathway from age 15 self-esteem to age 17 depressive symptoms (*B* = -0.041, *p* = .047; refer to Supplementary Table 15). This suggests that the associations between core beliefs and depression may depend on how depressive symptoms are operationalized. Most of previous studies on the associations between core beliefs and depression did not exclude self-esteem-related items from the depression measure (e.g., Study 2 in Ohannessian et al., 1999; Orth et al., 2008; 2018; van Tuijl et al., 2014; also refer to the meta-analytic work of Sowislo & Orth, 2013 for the common measures used in longitudinal studies on self-esteem and depression). However, one study conducted by Orth et al. (2008), which used a depression measure that did not overlap with self-esteem, supported the vulnerability theory but not the scar theory, which contradicted our findings. Because the approach of omitting conceptually overlapping items from the depression measure is not common, more work is needed to understand the impact of measurement on the associations between core beliefs and depression.

We also observed an intriguing and surprising finding – although the vulnerability theory was not supported by the multigroup model, the pathway from age 15 self-esteem to age 17 depressive symptoms was significant in the overall model where the groups are not separately analyzed (refer to Table 2). Specifically, the path coefficients decreased in size in the multigroup model as compared to the overall model, but the standard error of the coefficients remained similar. This may suggest a significant “vulnerability effect” introduced by between-group mean-level differences – that is, the cultural groups with higher group means on self-esteem also tend to show lower group means on later depressive symptoms. However, when analyzing individual level associations within each country, the data do not support an association between each adolescent’s earlier self-esteem and later depressive symptoms.

### Cross-cultural differences in the associations between core beliefs and depressive symptoms

Most of our findings were consistent across the 12 cultural contexts. However, a few cultural groups deviated from the general pattern. Only one cultural difference was directly related to our main hypotheses. Specifically, unlike the other groups in which the scar pathways were universally supported, in Sweden, age 15 depressive symptoms were unrelated to age 17 optimism. The finding for Sweden is surprising because it contradicts the literature that core beliefs are more strongly related to depression in individualistic cultures (e.g., Kwon & Kim, 2024; Smith et al., 2016; Stewart et al., 2004), although literature supporting such associations often only focuses on self-esteem or self-efficacy, rather than optimism. It is unclear what factors may be responsible for this finding. Potential explanations include the flourishing mental health system in Sweden (Forslund et al., 2020; Persson et al., 2017) as well as economic and cultural factors. Perhaps the therapeutic intervention and economic resources, as well as the individualistic values makes depression less relevant to externally focused beliefs. However, more work needs to be conducted to explore this possibility. Future work should aim to explore possible protective factors for this pathway (which may be operating in this cultural context) to inform treatment efforts for adolescent depression.

The other cultural differences that emerged in our model pertained to the stability of one variable (optimism), as well as the effects of the demographic variables on outcomes. One difference was the stability of age 15 optimism to age 17 optimism in the sample from Rome, Italy. Across groups, this pathway was significant and positive. In the sample from Rome, this pathway continued to be significant and positive – but the effect size was significantly larger than the other groups.

The four other differences we found across groups were related to gender and parental education. In the sample from Jordan, girls reported higher age 17 depressive symptoms, self-esteem, and optimism, and this pattern is also observed in the zero-order correlations. Across all other countries, gender was not a significant predictor of either self-esteem or optimism. Although speculative, one possible explanation may be that a strong emphasis on female education has developed in Jordan (Ajlouni et al., 2022; Khwaileh & Zaza, 2010). Academic achievements have been linked to improved self-esteem and optimism (Marques et al., 2017), so it is possible that this emphasis on female academic achievement is driving these findings. Across all other countries, girls reported significantly higher depressive symptoms, which is consistent with the literature (Shorey et al., 2022). However, this gender effect was significantly higher in Jordan.

The last country difference we found was in the Philippines where parental education, which we used as a measure of socioeconomic status, was predictive of age 17 optimism. This variable was not predictive of optimism in other countries. Most surprisingly, in the Philippines, higher parental education (and thus higher SES) was associated with *lower* optimism at age 17. One possible explanation for this association is that adolescents who have parents with more complete education are often met with higher expectations to succeed themselves (Sorbring & Lansford, 2019). However, due to the difficult economic context of the Philippines, success can often be difficult to secure regardless of educational opportunities. This pressure to succeed paired with a challenging context in which to succeed might manifest as decreased optimism for adolescents preparing to embark into adulthood.

### Strengths, limitations, and implications

The current study possesses many strengths, notably its cross-cultural design. Much research regarding how depressive symptoms and core beliefs are related longitudinally has been done in Western, industrialized countries. And no studies, in our knowledge, have examined these pathways in adolescence across multiple countries simultaneously. Our study fills this gap through a large and rigorous cross-cultural examination of these adolescent developmental pathways, using longitudinal data and controlling the effects of demographic factors. In addition, we were careful to exclude depressive symptom items that overlap with our core belief variables (i.e., feeling worthless), which enhanced the interpretability and validity of our findings, and suggested that the mixed findings in the literature may be due to different operationalizations of depression.

Despite these strengths, this study also has some limitations. The analysis was based on self-reports and could be subject to shared method effects. However, self-reports are also considered more reliable for accessing core beliefs and depressive symptoms, as adolescents themselves are the best informants of their own thoughts and feelings (Bowker et al., 2016). However, mental health awareness and stigma may vary widely across countries and ethnic groups, which may result in adolescents’ varying level of comfort and ability in reporting their symptoms. In addition, although the items for our measures were translated and evaluated for their cultural appropriateness, it remains possible that certain items could be interpreted differently or carry unique meanings across cultural contexts, which may influence our findings. Our findings need to be replicated in future studies using alternative informants and assessment methods (e.g., clinical assessment, and culturally based measures of depression and core beliefs). In addition, our sample also reported, on average, low depressive symptoms. Future studies need to examine these pathways in high-risk samples and inspect whether these pathways differ in adolescents experiencing higher, and perhaps clinical, levels of depressive symptoms. Furthermore, our study only included two time points, age 15 and age 17. Thus, we were unable to disentangle the within-person and between-person effects using more sophisticated models such as the Random Intercept Cross-Lagged Panel Model (RI-CLPM; Hamaker et al., 2015).

Overall, our findings have shown that adolescents’ depressive symptoms are linked to future core beliefs about the self and the social world, and such associations are largely universal across cultures. The findings highlight the detrimental longitudinal effects of adolescent depression on the formation of negative cognition patterns, as well as the urgent need to provide supportive resources for depressed adolescents across the globe. Future work should explore potential moderating mechanisms that improve the prognosis of depression, with a special focus on mitigating the impact on the negative belief patterns, as such belief patterns can have harmful effects on long-term outcomes for adolescents (Boden et al., 2008; Tetzner & Becker, 2025). Some work on potential moderators and mediators of the vulnerability pathways has been conducted (Sowislo & Orth, 2013), but very little work has focused on potential moderators of the scar pathway. Indeed, our deviational finding in the scar pathway in Sweden suggests the possibility of context-specific protective mechanisms that buffer the effects of the scar pathway, at least for externally focused core beliefs. Future work should aim to further explore the specific moderating mechanisms across diverse contexts. Such knowledge can inform prevention and treatment techniques to mitigate the insidious effects of adolescent psychopathology on future core beliefs.

## Supporting information

10.1017/S0954579425100497.sm001 Cortright et al. supplementary material Cortright et al. supplementary material

## Data Availability

The data and data analysis programing code used in the current study are available from the corresponding author upon reasonable request.

## Supplementary material

The supplementary material for this article can be found at https://doi.org/10.1017/S0954579425100497.
