# Supplementary material for: The relations among depressive symptoms, self-esteem, and optimism during adolescence: Longitudinal evidence from nine countries
Source: Dev Psychopathol. 2025 Sep 15:1–12. Online ahead of print. doi: 10.1017/S0954579425100497 (PMC12440366; doi:10.1017/S0954579425100497)
Supplement: Cortright et al. supplementary material [file S0954579425100497sup001.docx]

Table 1

*Descriptive Statistics and Zero-Order Correlations among Study Variables in Italy-Naples*

|  | 1 | 2 | 3 | 4 | 5 | 6 | 7 | 8 |
| --- | --- | --- | --- | --- | --- | --- | --- | --- |
| 1. Age 15 Depressive Symptoms | – | -.51^***^ | -.36^***^ | .69^***^ | -.44^***^ | -.42^***^ | .35^***^ | .06 |
| 2. Age 15 Self Esteem |  | – | .53^***^ | -.40^***^ | .59^***^ | .39^***^ | -.15 | -.22* |
| 3. Age 15 Optimism |  |  | – | -.42^***^ | .38^***^ | .61^***^ | -.01 | -.01 |
| 4. Age 17 Depressive Symptoms |  |  |  | – | -.47^***^ | -.51^***^ | .24* | .09 |
| 5. Age 17 Self Esteem |  |  |  |  | – | .66^***^ | -.10 | -.11 |
| 6. Age 17 Optimism |  |  |  |  |  | – | -.15 | -.11 |
| 7. Gender |  |  |  |  |  |  | – | .05 |
| 8. Highest Parental Education |  |  |  |  |  |  |  | – |
| *N* | 89 | 89 | 89 | 87 | 87 | 87 | 92 | 87 |
| Mean | 0.42 | 3.73 | 2.99 | 0.55 | 3.73 | 3.14 | 1.54 | 12.33 |
| *SD* | 0.36 | 0.90 | 1.02 | 0.44 | 0.87 | 1.02 | 0.50 | 4.72 |
| Minimum | 0.00 | 1.00 | 1.00 | 0.00 | 1.33 | 1.00 | 1.00 | 3.00 |
| Maximum | 1.67 | 5.00 | 5.00 | 1.67 | 5.00 | 5.00 | 2.00 | 24.00 |
| Skewness | 0.75 | -0.440 | 0.39 | 0.71 | -0.21 | -0.12 | -0.18 | 0.55 |
| Kurtosis | 0.38 | -0.04 | -0.77 | -0.45 | -0.39 | -0.69 | -2.01 | -0.18 |

^*^*p* ≤ .05. ^**^*p* ≤ .01. ^***^*p* ≤ .001.

Table 2

*Descriptive Statistics and Zero-Order Correlations among Study Variables in Italy-Rome*

|  | 1 | 2 | 3 | 4 | 5 | 6 | 7 | 8 |
| --- | --- | --- | --- | --- | --- | --- | --- | --- |
| 1. Age 15 Depressive Symptoms | – | -.46^***^ | -.19 | .60^***^ | -.26^**^ | -.24^*^ | .54^***^ | .03 |
| 2. Age 15 Self Esteem |  | – | .64^***^ | -.44^***^ | .61^***^ | .52^***^ | -.30^**^ | .06 |
| 3. Age 15 Optimism |  |  | – | -.30^**^ | .47^***^ | .63^***^ | -.10 | -.07 |
| 4. Age 17 Depressive Symptoms |  |  |  | – | -.49^***^ | -.39^***^ | .50*** | .17 |
| 5. Age 17 Self Esteem |  |  |  |  | – | .62^***^ | -.11 | -.02 |
| 6. Age 17 Optimism |  |  |  |  |  | – | -.17 | -.06 |
| 7. Gender |  |  |  |  |  |  | – | -.12 |
| 8. Highest Parental Education |  |  |  |  |  |  |  | – |
| *N* | 102 | 102 | 102 | 102 | 102 | 102 | 104 | 97 |
| Mean | 0.48 | 3.63 | 3.00 | 0.64 | 3.50 | 2.85 | 1.48 | 14.61 |
| *SD* | 0.41 | 0.78 | 0.92 | 0.44 | 0.87 | 1.02 | 0.50 | 4.49 |
| Minimum | 0.00 | 1.67 | 1.00 | 0.00 | 1.00 | 1.00 | 1.00 | 5.00 |
| Maximum | 1.83 | 5.00 | 5.00 | 1.83 | 5.00 | 5.00 | 2.00 | 28.00 |
| Skewness | 0.90 | -0.16 | 0.05 | 0.43 | -0.52 | 0.17 | 0.08 | 0.44 |
| Kurtosis | 0.74 | -0.35 | -0.80 | -0.35 | 0.18 | -0.81 | -2.03 | 0.14 |

^*^*p* ≤ .05. ^**^*p* ≤ .01. ^***^*p* ≤ .001.

Table 3

*Descriptive Statistics and Zero-Order Correlations among Study Variables in Jordan*

|  | 1 | 2 | 3 | 4 | 5 | 6 | 7 | 8 |
| --- | --- | --- | --- | --- | --- | --- | --- | --- |
| 1. Age 15 Depressive Symptoms | – | -.37^***^ | -.34^***^ | .40^***^ | -.01 | -.07 | .22^*^ | -.04 |
| 2. Age 15 Self Esteem |  | – | .60^***^ | -.01 | .33^***^ | .22^*^ | .28^**^ | -.11 |
| 3. Age 15 Optimism |  |  | – | -.07 | .32^***^ | .32^**^ | .26^**^ | -.06 |
| 4. Age 17 Depressive Symptoms |  |  |  | – | -.07 | -.24^*^ | .37^***^ | -.12 |
| 5. Age 17 Self Esteem |  |  |  |  | – | .66^***^ | .51^***^ | .04 |
| 6. Age 17 Optimism |  |  |  |  |  | – | .39^***^ | .17 |
| 7. Gender |  |  |  |  |  |  | – | .04 |
| 8. Highest Parental Education |  |  |  |  |  |  |  | – |
| *N* | 101 | 101 | 101 | 98 | 98 | 95 | 102 | 95 |
| Mean | 0.42 | 4.24 | 3.35 | 0.38 | 4.15 | 3.38 | 1.51 | 14.06 |
| *SD* | 0.44 | 0.78 | 1.01 | 0.42 | 0.85 | 1.01 | 0.50 | 2.39 |
| Minimum | 0.00 | 1.67 | 1.00 | 0.00 | 1.67 | 1.50 | 1.00 | 10.00 |
| Maximum | 1.83 | 5.00 | 5.00 | 1.83 | 5.00 | 5.00 | 2.00 | 20.00 |
| Skewness | 1.21 | -1.16 | 0.02 | 1.22 | -0.87 | 0.20 | -0.04 | 0.75 |
| Kurtosis | 0.79 | 1.21 | -0.97 | 0.91 | 0.32 | -1.07 | -2.04 | -0.08 |

^*^*p* ≤ .05. ^**^*p* ≤ .01. ^***^*p* ≤ .001.

Table 4

*Descriptive Statistics and Zero-Order Correlations among Study Variables in Sweden*

|  | 1 | 2 | 3 | 4 | 5 | 6 | 7 | 8 |
| --- | --- | --- | --- | --- | --- | --- | --- | --- |
| 1. Age 15 Depressive Symptoms | – | -.51^***^ | -.23 | .41^**^ | -.28^*^ | .08 | .32^**^ | -.01 |
| 2. Age 15 Self Esteem |  | – | .70^***^ | -.19 | .34^*^ | .20 | -.22 | .06 |
| 3. Age 15 Optimism |  |  | – | -.20 | .47^***^ | .44^***^ | -.14 | .16 |
| 4. Age 17 Depressive Symptoms |  |  |  | – | -.43^***^ | -.18 | .24 | -.08 |
| 5. Age 17 Self Esteem |  |  |  |  | – | .52^***^ | -.24 | .01 |
| 6. Age 17 Optimism |  |  |  |  |  | – | -.14 | .10 |
| 7. Gender |  |  |  |  |  |  | – | .02 |
| 8. Highest Parental Education |  |  |  |  |  |  |  | – |
| *N* | 73 | 72 | 73 | 62 | 65 | 65 | 84 | 46 |
| Mean | 0.51 | 3.83 | 3.08 | 0.51 | 3.94 | 3.39 | 1.60 | 16.65 |
| *SD* | 0.49 | 0.97 | 0.95 | 0.42 | 0.81 | 0.76 | 0.49 | 3.35 |
| Minimum | 0.00 | 1.33 | 1.25 | 0.00 | 2.00 | 1.75 | 1.00 | 9.00 |
| Maximum | 2.00 | 5.00 | 5.00 | 1.83 | 5.00 | 5.00 | 2.00 | 29.00 |
| Skewness | 1.10 | -0.67 | 0.13 | 0.89 | -0.57 | -0.18 | -0.40 | 0.73 |
| Kurtosis | 0.87 | -0.31 | -1.12 | 0.58 | -0.35 | -0.53 | -1.89 | 3.12 |

^*^*p* ≤ .05. ^**^*p* ≤ .01. ^***^*p* ≤ .001.

Table 5

*Descriptive Statistics and Zero-Order Correlations among Study Variables across All 12 Groups in Philippines*

|  | 1 | 2 | 3 | 4 | 5 | 6 | 7 | 8 |
| --- | --- | --- | --- | --- | --- | --- | --- | --- |
| 1. Age 15 Depressive Symptoms | – | -.39^***^ | -.25^*^ | .50^***^ | -.30^**^ | -.13 | .08 | .02 |
| 2. Age 15 Self Esteem |  | – | .56^***^ | -.21 | .34^**^ | .25^*^ | -.09 | -.09 |
| 3. Age 15 Optimism |  |  | – | -.21 | .36^***^ | .43^***^ | .05 | -.11 |
| 4. Age 17 Depressive Symptoms |  |  |  | – | -.50^***^ | -.39^***^ | .10 | .11 |
| 5. Age 17 Self Esteem |  |  |  |  | – | .66^***^ | -.05 | -.10 |
| 6. Age 17 Optimism |  |  |  |  |  | – | .07 | -.30^**^ |
| 7. Gender |  |  |  |  |  |  | – | .14 |
| 8. Highest Parental Education |  |  |  |  |  |  |  | – |
| *N* | 87 | 87 | 87 | 85 | 85 | 85 | 92 | 83 |
| Mean | 0.66 | 3.76 | 3.57 | 0.72 | 3.67 | 3.75 | 1.50 | 14.46 |
| *SD* | 0.44 | 0.86 | 0.89 | 0.45 | 0.80 | 0.89 | 0.50 | 4.05 |
| Minimum | 0.00 | 1.67 | 1.75 | 0.00 | 1.33 | 1.75 | 1.00 | 2.00 |
| Maximum | 2.00 | 5.00 | 5.00 | 1.67 | 5.00 | 5.00 | 2.00 | 26.00 |
| Skewness | 0.61 | -0.43 | -0.44 | 0.11 | -0.49 | -0.63 | 0.00 | -0.19 |
| Kurtosis | 0.11 | -0.30 | -1.01 | -1.10 | 0.31 | -0.41 | -2.05 | 1.68 |

^*^*p* ≤ .05. ^**^*p* ≤ .01. ^***^*p* ≤ .001.

Table 6

*Descriptive Statistics and Zero-Order Correlations among Study Variables across All 12 Groups in Kenya*

|  | 1 | 2 | 3 | 4 | 5 | 6 | 7 | 8 |
| --- | --- | --- | --- | --- | --- | --- | --- | --- |
| 1. Age 15 Depressive Symptoms | – | -.34^**^ | -.23^*^ | .47^***^ | -.08 | -.31^*^ | .10 | -.02 |
| 2. Age 15 Self Esteem |  | – | .17 | -.24 | .36^*^ | .20 | .03 | .01 |
| 3. Age 15 Optimism |  |  | – | -.23 | .17 | .19 | -.09 | -.00 |
| 4. Age 17 Depressive Symptoms |  |  |  | – | -.05 | -.33^*^ | .16 | .15 |
| 5. Age 17 Self Esteem |  |  |  |  | – | .15 | .13 | .04 |
| 6. Age 17 Optimism |  |  |  |  |  | – | -.11 | .03 |
| 7. Gender |  |  |  |  |  |  | – | .14 |
| 8. Highest Parental Education |  |  |  |  |  |  |  | – |
| *N* | 75 | 75 | 75 | 51 | 51 | 51 | 79 | 50 |
| Mean | 0.27 | 4.36 | 4.15 | 0.32 | 4.54 | 4.50 | 1.59 | 13.64 |
| *SD* | 0.29 | 0.68 | 0.89 | 0.29 | 0.53 | 0.59 | 0.49 | 3.44 |
| Minimum | 0.00 | 2.33 | 2.00 | 0.00 | 3.00 | 2.75 | 1.00 | 0.00 |
| Maximum | 1.00 | 5.00 | 5.00 | 1.00 | 5.00 | 5.00 | 2.00 | 20.00 |
| Skewness | 1.02 | -1.08 | -0.92 | 0.69 | -1.25 | -1.32 | -0.39 | -1.22 |
| Kurtosis | 0.03 | 0.46 | -0.23 | -0.36 | 0.86 | 1.26 | -1.89 | 4.03 |

^*^*p* ≤ .05. ^**^*p* ≤ .01. ^***^*p* ≤ .001.

Table 7

*Descriptive Statistics and Zero-Order Correlations among Study Variables in Thailand*

|  | 1 | 2 | 3 | 4 | 5 | 6 | 7 | 8 |
| --- | --- | --- | --- | --- | --- | --- | --- | --- |
| 1. Age 15 Depressive Symptoms | – | -.34^**^ | -.05 | .60^***^ | -.27^*^ | -.01 | .09 | .11 |
| 2. Age 15 Self Esteem |  | – | .39^***^ | -.32^**^ | .25^*^ | .16 | .02 | -.25^*^ |
| 3. Age 15 Optimism |  |  | – | -.15 | .11 | .33^**^ | -.02 | -.12 |
| 4. Age 17 Depressive Symptoms |  |  |  | – | -.50^***^ | -.21^*^ | .11 | -.04 |
| 5. Age 17 Self Esteem |  |  |  |  | – | .35^***^ | .05 | .07 |
| 6. Age 17 Optimism |  |  |  |  |  | – | .05 | .112 |
| 7. Gender |  |  |  |  |  |  | – | -.09 |
| 8. Highest Parental Education |  |  |  |  |  |  |  | – |
| *N* | 83 | 83 | 83 | 89 | 89 | 89 | 90 | 69 |
| Mean | 0.46 | 3.88 | 3.57 | 0.71 | 3.70 | 3.77 | 1.51 | 14.19 |
| *SD* | 0.37 | 0.70 | 0.74 | 0.42 | 0.75 | 0.91 | 0.50 | 5.23 |
| Minimum | 0.00 | 2.00 | 2.00 | 0.00 | 1.33 | 1.25 | 1.00 | 4.00 |
| Maximum | 1.50 | 5.00 | 5.00 | 1.83 | 5.00 | 5.00 | 2.00 | 25.00 |
| Skewness | 0.59 | -0.25 | -0.03 | 0.36 | -0.40 | -0.53 | -0.05 | -0.11 |
| Kurtosis | -0.36 | -0.28 | -0.42 | -0.33 | 0.31 | -0.30 | -2.04 | -0.62 |

^*^*p* ≤ .05. ^**^*p* ≤ .01. ^***^*p* ≤ .001.

Table 8

*Descriptive Statistics and Zero-Order Correlations among Study Variables in United States (African American)*

|  | 1 | 2 | 3 | 4 | 5 | 6 | 7 | 8 |
| --- | --- | --- | --- | --- | --- | --- | --- | --- |
| 1. Age 15 Depressive Symptoms | – | -.56^***^ | -.44^***^ | .50^**^ | -.31^**^ | -.32^*^ | .34^**^ | .11 |
| 2. Age 15 Self Esteem |  | – | .55^***^ | -.24^*^ | .35^**^ | .40^**^ | -.07 | -.02 |
| 3. Age 15 Optimism |  |  | – | -.19 | .24^*^ | .38^***^ | -.08 | -.06 |
| 4. Age 17 Depressive Symptoms |  |  |  | – | -.47^***^ | -.37^**^ | .47^***^ | .09 |
| 5. Age 17 Self Esteem |  |  |  |  | – | .56^***^ | -.26^*^ | .06 |
| 6. Age 17 Optimism |  |  |  |  |  | – | -.11 | .02 |
| 7. Gender |  |  |  |  |  |  | – | .26^*^ |
| 8. Highest Parental Education |  |  |  |  |  |  |  | – |
| *N* | 88 | 86 | 87 | 71 | 72 | 72 | 89 | 73 |
| Mean | 0.32 | 4.39 | 3.66 | 0.43 | 4.25 | 3.78 | 1.51 | 14.23 |
| *SD* | 0.38 | 0.81 | 0.98 | 0.49 | 0.95 | 1.00 | 0.50 | 4.15 |
| Minimum | 0.00 | 2.00 | 1.25 | 0.00 | 1.00 | 1.50 | 1.00 | 2.00 |
| Maximum | 1.83 | 5.00 | 5.00 | 1.83 | 5.00 | 5.00 | 2.00 | 30.00 |
| Skewness | 1.36 | -1.44 | -0.34 | 1.08 | -1.60 | -0.48 | -0.02 | 0.26 |
| Kurtosis | 1.77 | 1.28 | -0.75 | 0.22 | 2.31 | -0.78 | -2.05 | 3.92 |

^*^*p* ≤ .05. ^**^*p* ≤ .01. ^***^*p* ≤ .001.

Table 9

*Descriptive Statistics and Zero-Order Correlations among Study Variables in United States (European American)*

|  | 1 | 2 | 3 | 4 | 5 | 6 | 7 | 8 |
| --- | --- | --- | --- | --- | --- | --- | --- | --- |
| 1. Age 15 Depressive Symptoms | – | -.66^***^ | -.45^***^ | .51^***^ | -.43^***^ | -.38^***^ | .23^*^ | -.04 |
| 2. Age 15 Self Esteem |  | – | .56^***^ | -.43^***^ | .61^***^ | .47^***^ | -.07 | .14 |
| 3. Age 15 Optimism |  |  | – | -.36^**^ | .49^***^ | .64^***^ | -.05 | .04 |
| 4. Age 17 Depressive Symptoms |  |  |  | – | -.68^***^ | -.49^***^ | .20 | .21 |
| 5. Age 17 Self Esteem |  |  |  |  | – | .71^***^ | .03 | .03 |
| 6. Age 17 Optimism |  |  |  |  |  | – | -.01 | -.04 |
| 7. Gender |  |  |  |  |  |  | – | .02 |
| 8. Highest Parental Education |  |  |  |  |  |  |  | – |
| *N* | 89 | 88 | 88 | 77 | 78 | 78 | 93 | 77 |
| Mean | 0.54 | 3.94 | 3.26 | 0.69 | 3.77 | 3.13 | 1.41 | 18.13 |
| *SD* | 0.50 | 0.96 | 1.02 | 0.50 | 1.08 | 1.10 | 0.49 | 4.61 |
| Minimum | 0.00 | 1.00 | 1.00 | 0.00 | 1.00 | 1.00 | 1.00 | 4.00 |
| Maximum | 1.83 | 5.00 | 5.00 | 2.00 | 5.00 | 5.00 | 2.00 | 28.00 |
| Skewness | 0.97 | -1.04 | -0.08 | 0.42 | -0.71 | 0.03 | 0.38 | -0.61 |
| Kurtosis | 0.09 | 0.60 | -0.88 | -0.62 | -0.35 | -1.02 | -1.90 | 1.42 |

^*^*p* ≤ .05. ^**^*p* ≤ .01. ^***^*p* ≤ .001.

Table 10

*Descriptive Statistics and Zero-Order Correlations among Study Variables in United States (Hispanic)*

|  | 1 | 2 | 3 | 4 | 5 | 6 | 7 | 8 |
| --- | --- | --- | --- | --- | --- | --- | --- | --- |
| 1. Age 15 Depressive Symptoms | – | -.52^***^ | -.32^*^ | .53^***^ | -.27 | -.20 | .31^*^ | .24 |
| 2. Age 15 Self Esteem |  | – | .66^***^ | -.31^*^ | .60^***^ | .53^***^ | .12 | -.15 |
| 3. Age 15 Optimism |  |  | – | -.06 | .39^**^ | .45^***^ | .08 | -.14 |
| 4. Age 17 Depressive Symptoms |  |  |  | – | -.52^***^ | -.25 | .08 | .13 |
| 5. Age 17 Self Esteem |  |  |  |  | – | .70^***^ | .10 | -.18 |
| 6. Age 17 Optimism |  |  |  |  |  | – | .17 | -.25 |
| 7. Gender |  |  |  |  |  |  | – | .15 |
| 8. Highest Parental Education |  |  |  |  |  |  |  | – |
| *N* | 62 | 63 | 63 | 61 | 61 | 61 | 70 | 57 |
| Mean | 0.33 | 4.12 | 3.55 | 0.44 | 4.16 | 3.87 | 1.50 | 11.11 |
| *SD* | 0.39 | 0.74 | 0.96 | 0.44 | 0.81 | 0.89 | 0.50 | 4.07 |
| Minimum | 0.00 | 2.33 | 1.25 | 0.00 | 1.67 | 1.50 | 1.00 | 3.00 |
| Maximum | 1.83 | 5.00 | 5.00 | 1.83 | 5.00 | 5.00 | 2.00 | 22.00 |
| Skewness | 1.60 | -0.63 | -0.20 | 1.08 | -0.79 | -0.57 | 0.00 | 0.41 |
| Kurtosis | 2.97 | -0.51 | -0.61 | 0.84 | 0.13 | -0.49 | -2.06 | 0.55 |

^*^*p* ≤ .05. ^**^*p* ≤ .01. ^***^*p* ≤ .001.

Table 11

*Descriptive Statistics and Zero-Order Correlations among Study Variables in Colombia*

|  | 1 | 2 | 3 | 4 | 5 | 6 | 7 | 8 |
| --- | --- | --- | --- | --- | --- | --- | --- | --- |
| 1. Age 15 Depressive Symptoms | – | -.41^***^ | -.30^**^ | .62^***^ | -.31^**^ | -.25^*^ | .43^***^ | .04 |
| 2. Age 15 Self Esteem |  | – | .47^***^ | -.27^*^ | .36^**^ | .26^*^ | -.09 | .06 |
| 3. Age 15 Optimism |  |  | – | -.19 | .41^***^ | .43^***^ | -.09 | .10 |
| 4. Age 17 Depressive Symptoms |  |  |  | – | -.41^***^ | -.34^**^ | .40^***^ | .04 |
| 5. Age 17 Self Esteem |  |  |  |  | – | .59^***^ | .05 | -.04 |
| 6. Age 17 Optimism |  |  |  |  |  | – | -.03 | .12 |
| 7. Gender |  |  |  |  |  |  | – | .07 |
| 8. Highest Parental Education |  |  |  |  |  |  |  | – |
| *N* | 80 | 80 | 80 | 79 | 79 | 79 | 80 | 79 |
| Mean | 0.50 | 3.96 | 3.80 | 0.47 | 4.16 | 3.94 | 1.51 | 12.20 |
| *SD* | 0.42 | 0.77 | 0.76 | 0.43 | 0.66 | 0.73 | 0.50 | 4.95 |
| Minimum | 0.00 | 1.00 | 1.75 | 0.00 | 2.67 | 1.50 | 1.00 | 1.00 |
| Maximum | 1.67 | 5.00 | 5.00 | 1.67 | 5.00 | 5.00 | 2.00 | 25.00 |
| Skewness | 0.86 | -0.89 | -0.63 | 0.64 | -0.41 | -0.95 | -0.05 | 0.07 |
| Kurtosis | 0.26 | 1.87 | -0.05 | -0.60 | -0.51 | 1.22 | -2.05 | -0.37 |

^*^*p* ≤ .05. ^**^*p* ≤ .01. ^***^*p* ≤ .001.

Table 12

*Descriptive Statistics and Zero-Order Correlations among Study Variables in China (Chongqing)*

|  | 1 | 2 | 3 | 4 | 5 | 6 | 7 | 8 |
| --- | --- | --- | --- | --- | --- | --- | --- | --- |
| 1. Age 15 Depressive Symptoms | – | -.32^***^ | -.18 | .50^***^ | -.23^*^ | -.21^*^ | .19^*^ | -.03 |
| 2. Age 15 Self Esteem |  | – | .59^***^ | -.41^***^ | .55^***^ | .33^***^ | -.00 | .19 |
| 3. Age 15 Optimism |  |  | – | -.12 | .31^**^ | .34^***^ | -.05 | .16 |
| 4. Age 17 Depressive Symptoms |  |  |  | – | -.62^***^ | -.29^**^ | .17 | -.25^*^ |
| 5. Age 17 Self Esteem |  |  |  |  | – | .48^***^ | -.03 | .22^*^ |
| 6. Age 17 Optimism |  |  |  |  |  | – | -.07 | .19 |
| 7. Gender |  |  |  |  |  |  | – | -.21 |
| 8. Highest Parental Education |  |  |  |  |  |  |  | – |
| *N* | 113 | 113 | 113 | 95 | 97 | 97 | 113 | 84 |
| Mean | 0.59 | 3.35 | 3.09 | 0.68 | 3.58 | 3.08 | 1.52 | 12.38 |
| *SD* | 0.44 | 0.90 | 0.98 | 0.52 | 0.96 | 1.01 | 0.50 | 3.41 |
| Minimum | 0.00 | 1.00 | 1.00 | 0.00 | 1.00 | 1.00 | 1.00 | 6.00 |
| Maximum | 1.67 | 5.00 | 5.00 | 1.83 | 5.00 | 5.00 | 2.00 | 21.00 |
| Skewness | 0.55 | 0.07 | 0.37 | 0.39 | -0.64 | -0.04 | -0.09 | 0.39 |
| Kurtosis | -0.23 | -0.21 | -0.44 | -0.84 | 0.14 | -0.69 | -2.03 | -0.66 |

^*^*p* ≤ .05. ^**^*p* ≤ .01. ^***^*p* ≤ .001.

Table 13

*Reliability for All Variables Total and By Group*

|  | Group | | | | | | | | | | | | |
| --- | --- | --- | --- | --- | --- | --- | --- | --- | --- | --- | --- | --- | --- |
| Variable | All Groups | Italy (Naples) | Italy (Rome) | Kenya | Philippines | Thailand | Sweden | USA (AA) | USA (EA) | USA (H) | Colombia | Jordan | China |
| Age 15 Depression | .79 | .74 | .76 | .62 | .77 | .76 | .84 | .79 | .85 | .80 | .78 | .81 | .78 |
| Age 15  Self Esteem | .84 | .89 | .82 | .48 | .80 | .78 | .90 | .87 | .91 | .81 | .78 | .79 | .81 |
| Age 15 Optimism | .86 | .86 | .86 | .79 | .87 | .75 | .85 | .88 | .87 | .89 | .83 | .87 | .78 |
| Age 17 Depression | .80 | .79 | .75 | .60 | .76 | .76 | .78 | .86 | .82 | .82 | .80 | .81 | .85 |
| Age 17  Self Esteem | .83 | .83 | .82 | .43 | .71 | .81 | .83 | .86 | .91 | .86 | .74 | .83 | .83 |
| Age 17 Optimism | .87 | .88 | .89 | .71 | .85 | .86 | .66 | .85 | .91 | .83 | .80 | .91 | .83 |

*Notes.* Due to the low reliability scores for some of the variables in our sample from Kenya, we also conducted our analyses excluding this group. Our results did not significantly change by excluding the sample from Kenya. Thus, we decided to include the sample from Kenya in our final analyses.

Table 14

*R Squares for All Dependent Variables Across the 12 Groups*

|  | Group | | | | | | | | | | | |
| --- | --- | --- | --- | --- | --- | --- | --- | --- | --- | --- | --- | --- |
| Dependent Variable | Italy (Naples) | Italy (Rome) | Kenya | Philippines | Thailand | Sweden | USA (AA) | USA (EA) | USA (H) | Colombia | Jordan | China |
| Age 17 Depression | 0.32^***^ | 0.36^***^ | 0.31^***^ | 0.28^***^ | 0.26^***^ | 0.36^***^ | 0.25^***^ | 0.31^***^ | 0.27^***^ | 0.35^***^ | 0.35^***^ | 0.23^***^ |
| Age 17  Self Esteem | 0.25^***^ | 0.22^***^ | 0.24^***^ | 0.23^***^ | 0.15^***^ | 0.30^***^ | 0.17^***^ | 0.23^***^ | 0.25^***^ | 0.26^***^ | 0.38^***^ | 0.20^***^ |
| Age 17  Optimism | 0.23^***^ | 0.38^***^ | 0.25^***^ | 0.26^***^ | 0.11^***^ | 0.25^***^ | 0.19^***^ | 0.23^***^ | 0.24^***^ | 0.22^***^ | 0.28^***^ | 0.18^***^ |

^*^*p* ≤ .05. ^**^*p* ≤ .01. ^***^*p* ≤ .001.

Table 15

*Predictions of Age 17 Depressive Symptoms, Self-Esteem, and Optimism from the Same Set of Variables at Age 15 and Covariates, Using All Depression-Relevant Items in CBCL (i.e., Including the Depression Items Overlapping with Core Beliefs)*

|  |  | 1. Overall Model across Groups and Countries | | | |  | 2. Final Multigroup Model by Groups and Countries^a^ | | |
| --- | --- | --- | --- | --- | --- | --- | --- | --- | --- |
| DV | Predictor | *B* | *SE* | *p* | *R^2^* |  | *B* | *SE* | *p* |
| Age 17 Depressive Symptoms | Age 15 Depressive Symptoms | **0.500** | **0.038** | **<.001** |  |  | **0.502** | **0.037** | **<.001** |
|  | Age 15 Self-Esteem | **-0.040** | **0.020** | **.050** |  |  | **-0.041** | **0.020** | **.047** |
|  | Age 15 Optimism | -0.023 | 0.016 | .157 |  |  | -0.023 | 0.016 | .152 |
|  | Gender (1=Boy, 2=Girl) | **0.102** | **0.024** | **<.001** |  |  | **0.101** | **0.023** | **<.001** |
|  | Parental Education | 0.003 | 0.003 | .375 |  |  | 0.003 | 0.003 | .354 |
|  |  |  |  |  | **.351** |  |  |  |  |
| Age 17 Self- Esteem | Age 15 Depressive Symptoms | **-0.209** | **0.079** | **.008** |  |  | **-0.223** | **0.078** | **.004** |
|  | Age 15 Self-Esteem | **0.338** | **0.047** | **<.001** |  |  | **0.328** | **0.047** | **<.001** |
|  | Age 15 Optimism | **0.122** | **0.033** | **<.001** |  |  | **0.115** | **0.033** | **<.001** |
|  | Gender (1=Boy, 2=Girl) | **0.099** | **0.050** | **.045** |  |  | 0.043 | 0.051 | .392 |
|  |  |  |  |  |  |  | **(0.783** | **0.136** | **<.001)^b^** |
|  | Parental Education | 0.003 | 0.006 | .563 |  |  | 0.003 | 0.006 | .621 |
|  |  |  |  |  | **.296** |  |  |  |  |
| Age 17 Optimism | Age 15 Depressive Symptoms | -0.155 | 0.085 | .067 |  |  | **-0.222** | **0.086** | **.010** |
|  |  |  |  |  |  |  | **(0.204** | **0.102** | **.045)^c^** |
|  | Age 15 Self-Esteem | **0.130** | **0.047** | **.006** |  |  | **0.121** | **0.047** | **.009** |
|  | Age 15 Optimism | **0.365** | **0.040** | **<.001** |  |  | **0.352** | **0.039** | **<.001** |
|  | Gender (1=Boy, 2=Girl) | 0.064 | 0.055 | .239 |  |  | 0.027 | 0.055 | .631 |
|  |  |  |  |  |  |  | **(0.687** | **0.171** | **<.001)^b^** |
|  | Parental Education | -0.001 | 0.007 | .937 |  |  | 0.005 | 0.007 | .487 |
|  |  |  |  |  | **.289** |  | **(-0.191** | **0.057** | **.001)** ^d^ |

*Notes.* *B* = Unstandardized path coefficient. *SE* = Standard error of the unstandardized coefficient. We present the final multigroup model in the table, which fit the data well: χ^2^(160) = 176.56, *p* = .18; CFI = .992; RMSEA = .034, SRMR = .10. We report the country/group-invariant paths and put country/group-specific paths in the parentheses with the country/group noted. Shaded cells represent the country/group-specific paths. Significant findings are bolded. ^a^Jordan. ^b^Sweden. ^c^Rome, Italy. ^d^Philippines.
